# A Candidate Antigen of the Recombinant Membrane Protein Derived from the Porcine Deltacoronavirus Synthetic Gene to Detect Seropositive Pigs

**DOI:** 10.3390/v15051049

**Published:** 2023-04-25

**Authors:** Francisco Jesus Castañeda-Montes, José Luis Cerriteño-Sánchez, María Azucena Castañeda-Montes, Julieta Sandra Cuevas-Romero, Susana Mendoza-Elvira

**Affiliations:** 1 Centro Nacional de Investigación Disciplinaria en Salud Animal e Inocuidad, Instituto Nacional de Investigaciones Forestales, Agrícolas y Pecuarias, Km 15.5 Carretera México-Toluca, Palo Alto, Cuajimalpa, Ciudad de México 05110, Mexico; fjcastmont@gmail.com (F.J.C.-M.); cerriteno.jose@inifap.gob.mx (J.L.C.-S.); azucena.castaneda.montes@gmail.com (M.A.C.-M.); 2 Posgrado en Ciencias de la Producción y de la Salud Animal, Facultad de Estudios Superiores Cuautitlán, Estado de México, Universidad Nacional Autónoma de México, Ciudad de México 04510, Mexico

**Keywords:** PDCoV, recombinant protein, M protein, iELISA, antigen

## Abstract

Porcine deltacoronavirus (PDCoV) is an emergent swine coronavirus which infects cells from the small intestine and induces watery diarrhea, vomiting and dehydration, causing mortality in piglets (>40%). The aim of this study was to evaluate the antigenicity and immunogenicity of the recombinant membrane protein (M) of PDCoV (*r*M-PDCoV), which was developed from a synthetic gene obtained after an in silico analysis with a group of 138 GenBank sequences. A 3D model and phylogenetic analysis confirmed the highly conserved M protein structure. Therefore, the synthetic gene was successfully cloned in a pETSUMO vector and transformed in *E. coli* BL21 (DE3). The *r*M-PDCoV was confirmed by SDS-PAGE and Western blot with ~37.7 kDa. The *r*M-PDCoV immunogenicity was evaluated in immunized (BLAB/c) mice and iELISA. The data showed increased antibodies from 7 days until 28 days (*p* < 0.001). The *r*M-PDCoV antigenicity was analyzed using pig sera samples from three states located in “El Bajío” Mexico and positive sera were determined. Our results show that PDCoV has continued circulating on pig farms in Mexico since the first report in 2019; therefore, the impact of PDCoV on the swine industry could be higher than reported in other studies.

## 1. Introduction

Porcine deltacoronavirus (PDCoV) was the last swine coronavirus (SCoV) to be discovered and is considered an emergent virus. After the first isolation (HKU15) of PDCoV in China in 2012 [1], there have been several reports of detection in both Asia and America [2,3,4,5,6,7,8]. The pathogenesis of the virus preferentially occurs within the small intestine’s enterocytes, where the virus interacts with its antigens and their receptors, facilitating their invasion and proliferation into them [9]. Damage to the epithelia is traduced in watery diarrhea, dehydration and vomiting signs that easily affect piglets, with mortality rates of >40%, causing serious economic losses worldwide [10,11,12,13]. PDCoV frequently co-infects with porcine epidemic diarrhea virus (PEDV), transmissible gastroenteritis virus (TGEV) and rotavirus C, increasing intestinal cell damage and inducing a severe infection [14,15,16]. In this scenario, an early and suitable diagnosis is of vital importance.

PDCoV has a positive single-stranded RNA genome with an approximate length of 25 kb. The genome organization is in the following order: 5′ untranslated region (UTR), open reading frame 1a/1b (ORF1a/1b), spike (S), envelope (E), membrane (M), nonstructural protein 6 (NS6), nucleocapsid (N), nonstructural protein 7 (NS7) and 3′ UTR [17,18]. The coronavirus M protein shares the same structural characteristics as ~230 amino acids in length and is a type III transmembrane glycoprotein composed of a short N-terminal domain situated outside the virion membrane, three transmembrane domains and a carboxy-terminal domain situated inside the particle [19,20]. The M protein is located among the S protein in the virus envelope, interacts with small amounts of E and is the primary driver of the virus budding process [21].

The M protein is implicated in multiple viral functions; it is the most abundant viral structural protein [22] with lower genetic variability (highly conserved) compared to the S sequence [23]. Interaction between the S protein and the M protein is necessary for retaining S in the ER-Golgi intermediate compartment (ERGIC)/Golgi complex and its incorporation into new virions, but it is unnecessary for the assembly process [24]. M and N proteins joined together to stabilize the N protein–RNA complex and the internal core of virions, promoting the viral assembly’s end [25,26,27,28]. Moreover, M and E proteins compose the viral envelope and their interaction is sufficient for producing and releasing viral particles [24,29,30].

Antigenically, the M protein promotes the production of antibodies with virus-neutralizing activity [31]. It has been observed that the M protein of TGEV acts as a dominant immunogen promoting the production of monoclonal antibodies [32]. In PEDV, the M protein has shown highly conserved epitopes among different PEDV strains, which elicit the formation of protective antibodies [33]. In coronaviruses that infect humans, such as SARS-CoV, the M protein holds dominant cellular immunogenicity, which can stimulate the response of T and B-cells with immunodominant epitopes [34,35,36]. The M protein of a cat coronavirus, such as feline infectious peritonitis virus (FIPV), can promote the response of Th1 cells by producing immunodominant antibodies [37]. Likewise, a protein M epitope of avian infectious bronchitis coronavirus (IBV) can elicit the production of B-cell monoclonal antibodies [38]. Furthermore, the cross-reactivity of PDCoV with antibodies to either the PEDV or TGEV M protein has not been previously observed [9,39,40]. Therefore, the PEDV M protein could be a good target for antigen development [41].

This study deals with the construction and expression of a recombinant protein from a synthetic gene in *E. coli* BL21 (DE3), obtained after in silico analysis from 138 sequences of the M protein of PDCoV (M-PDCoV). The immunogenicity of recombinant M protein was evaluated using experimental BLB/c mice groups. Then, the antigenicity was evaluated using sera samples from pig farms located in three Mexican states: Guanajuato, Aguascalientes and Jalisco. These states are located in a region called “El Bajío”, which is considered to be one of the main swine producer regions in Mexico.

## 2. Materials and Methods

### 2.1. In Silico Analysis of M-PDCoV

A total of 138 M-PDCoV sequences available in GenBank were obtained from China, Laos, Japan, Vietnam, Thailand and the USA, dating from 2012 to 2019 (Table S1). To determine phylogenetic relationships among the 138 sequences, a maximum likelihood phylogenetic tree using RAxML v8.0 was made [42]. The sequences were aligned using a Clustal Omega online server [43]. RAxML was run, using an HIVb amino acid substitution model [44] calculated by ProtTest software [45] and 1000 bootstraps were evaluated to assess support values. Moreover, a consensus sequence of 217 amino acids was obtained using Jalview software [46]. Potential antigenicity sites were predicted with the Jameson-Wolf method using Protean, DNAStar v17.2 [47]. Hydrophobic and hydrophilic sites were predicted with Kyte and Doolittle and Emini et al.’s methods using Protean, DNAStar v17.2 [48,49]. Additionally, predicted tertiary structures for the consensus sequence, HKU15 (GenBank: JQ065042) and CHzmd2019 (GenBank: MN781985) were determined using I-TASSER [50]. The M protein predicted tertiary structures of the PEDV CV777 (GenBank: AAK38659) and TGEV Miller M6 (GenBank: ABG89300) were included for comparison. I-TASSER predicted 5 possible structural models. The best quality predicted model was selected according to a C-score value. The C-score value is calculated based on the significance of threading template alignments and the convergence parameters of the structure assembly simulations, and range from −5 to 2, where a higher value signifies a model with high confidence. A predicted model whit a C-score >−1.5 usually has a correct fold [51,52]. The predicted models were visualized using the PyMOL Molecular Graphics System, Version 2.0 Schrödinger, LLC software. Moreover, the template modeling score (TM-score) was determined to assess the topological similarities and ranged from 0 to 1, where 1 indicates a perfect match between two structures [53]. The TM-score value was determined for the consensus sequence, HKU15 (GenBank: JQ065042), CHzmd2019 (GenBank: MN781985), PEDV CV777 (GenBank: AAK38659) and TGEV Miller M6 (GenBank: ABG89300). Lastly, the amino acid identity (AAI) was determined to analyze the consensus sequence, HKU15 (GenBank: JQ065042), CHzmd2019 (GenBank: MN781985) PEDV CV777 (GenBank: AAK38659) and TGEV Miller M6 (GenBank: ABG89300). AAI was determined using the online Sequence Manipulation Suite (SMS) [54]. NetNGlyc-1.0 online software was used to determine potential glycosylated sites in the consensus sequence.

### 2.2. Molecular Cloning and Expression of M-PDCoV in a pETSUMO Plasmid 

A synthetic gene (654 bp) from the consensus amino acid sequence was obtained from a commercial supplier and verified using the following primer sequences: Fw 5′-GACGCAGAAGAGTGGCAAATTATT-3′ and Rev 5′-GCGCTACTACATATACTTATACAGGCG-3. The synthetic gene was cloned in the pETSUMO plasmid and positive recombinant were transformed into *E. coli* BL21 (DE3) cells. Transformants cells were selected in LB agar plates supplemented with kanamycin, 50 µg/mL and used to purify the plasmid using a Wizard^®^ Plus SV Miniprep kit (Promega). The clones were confirmed by PCR using the sequences of the Fw primer mentioned above and the Rev T7 primer: 5′-TAGTTATTGCTCAGCGGTGG-3′. The recombinant M-PDCoV (*r*M-PDCoV) expression in *E. coli* BL21 (DE3) cells and protein purification was made according to the Lara-Romero protocol [55]. The purified recombinant protein was separated by 12% SDS-PAGE and confirmed by Western blot, and the concentration was determined according to the Bradford method [56]. Briefly, the nitrocellulose membranes used in WB were blocked with 5% nonfat milk in a TBS-Tween buffer (20 mM Tris-HCl, pH 8, 0.15 M NaCl and 0.05% Tween 20) at 4 °C for 1 h with moderate agitation. The blocked membranes were washed with TBS-Tween buffer and incubated with anti-histidine (diluted to 1:5000) as the primary antibody and a mouse anti-IgG conjugated with horseradish peroxidase (dilution 1:5000) as the secondary antibody (1:5000). Protein bands were visualized with a DAB ™ substrate (3,3′-diaminobenzidine tetrahydrochloride) (Sigma-Aldrich, St. Louis, MO, USA) with 10 mL of development solution (PBS, 12 mg of DAB and 300 μL 3.4% H_2_O_2_).

### 2.3. BALB/c Mice Immunization

The immunogenicity of *r*M-PDCoV was evaluated by mice immunization using two experimental groups and a control group. Each group consisted of 8 BALB/c mice who were 28 days old. One experimental group was immunized with 5 µg/mouse of the *r*M-PDCoV protein diluted in PBS buffer, 1X pH 7.4. A second group immunized with 5 µg/mouse formulated with immuno-stimulating complex, Matrix-M^TM^ as an adjuvant (Isconova AB, Uppsala, Sweden) [57]. The control group was only immunized with 200 µL of PBS buffer 1X pH 7.4. The final volume dose was 200 µL and two doses were applied subcutaneously on day 1 and day 14, respectively. The blood sample was collected through the caudal vein on days 7, 14, 21 and 28. The production of antibodies was evaluated by indirect ELISA.

To analyze the *r*M-PDCoV antigenicity, an iELISA assay was carried out using the serum samples obtained from the mice. Briefly, 75 ng of purified protein was absorbed per well in a microplate and subsequently incubated with mice sera samples (diluted to 1:150). A secondary antibody (mouse anti-IgG-HRP) was used with a dilution of 1:20,000. The chromogenic reaction was developed using 3,3′,5,5-tetramethylbenzidine (TMB) substrates (Sigma-Aldrich, St. Louis, MO, USA), as previously described [58,59] and measured at an absorbance of 450 nm. Statistical analyses were performed using two-way ANOVA to compare immunized groups with protein plus adjuvant versus protein alone on different days. Differences were calculated using KlusKal Wallis and Dunn`s test to calculate the *p*-value, the statistically significant was considered with a 95% confidence interval, (* *p* < 0.05; ** *p* < 0.005) and graphs were constructed using SigmaPlot version 12.5 (Systat Software Inc., San Jose, CA, USA). All summary data are presented as means ± standard error of mean (SEM).

At the end of the experiment, the mice were euthanized using a CO_2_ chamber following the ethical procedure of the NOM-062-ZOO-1999; SAGARPA. The animals were handled at the house facility of the National Microbiology Research Centre (CENID-SAI), Instituto Nacional de Investigaciones Forestales, Agrícolas y Pecuarias, INIFAP.

### 2.4. Antigenicity Evaluation Using Pig Farm Sera Samples by Indirect ELISA Assay

An iELISA was performed according to J. S. Cuevas-Romero et al [59]. Briefly, to standardize the ELISA assay, different concentrations of antigen (25, 50 and 75 ng), dilutions of the sera sample (1:200, 1:300, 1:500, 1:600, 1:800 and 1:1000) and dilutions of the secondary antibody were evaluated (1:10,000, 1:12,000, 1:15,000 and 1:20,000), and 75 ng of antigen, 1:1000 diluted sera and 1:20,000 diluted secondary antibody were the optimal conditions to perform the test. Then, 96-well flat-bottom plates was sensitized with 75 ng of *r*M-PDCoV. Positive (*n* = 8) and negative (*n* = 9) control sera were from a pig naturally infected with PDCoV and a non-infected pig from a pathogen free farm, both sera controls confirmed by a Western blot analysis (Supplementary Figure S1). A horseradish peroxidase (HRP)-labeled anti-pig-IgG (Sigma-Aldrich, St. Louis, MO, USA), diluted to 1:20,000, was used as the secondary antibody. The chromogenic reaction was developed using TMB (Sigma-Aldrich, St. Louis, MO, USA) substrate. Results were graphed using the Sigma plot 12.5 program. To determinate the cut-off value, 37 sera samples from a pathogen-free pig farm, without infection outbreaks and without vaccination history, were used. These sera were previously confirmed for the absence of PDCoV by Western blot. Absorbance was averaged and the value of 3 standard deviations was considered to obtain the absorbance. Then, 44 sera samples were taken from pig farms located in 3 Mexican states: Guanajuato (*n* = 24), Aguascalientes (*n* = 9) and Jalisco (*n* = 11). These states are located in the “El Bajío” region, which is considered to be one of the main swine producer regions in Mexico. The highest absorbance was considered to have 100% positivity (PP). Positivity was determined for 44 sera samples. The result was graphed using SigmaPlot version 12.5 (Systat Software Inc., San Jose, CA, USA).

## 3. Results

### 3.1. In Silico Analysis

**Figure 1:**
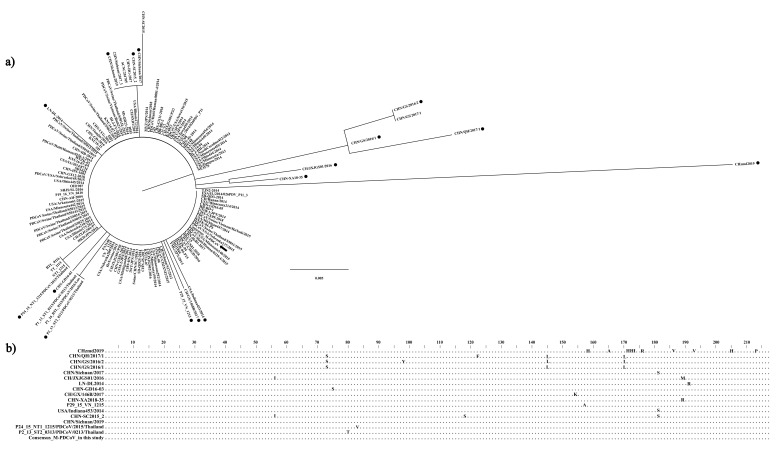
Phylogenetic tree. (**a**) Maximum likelihood phylogenetic tree displaying the 138 PDCoV protein M sequences available in Gen Bank. The black arrow shows the consensus M-PDCoV used in this study to develop a recombinant protein. The black dots show the selection of 16 sequences with branch length differences. (**b**) A comparison of the 16 sequences showed differences at the amino acid level. The sequence CHzm2019 from China showed the longest branch in the phylogeny and showed 10 different amino acids.

A total of 138 M-PDCoV sequences (Table S1) with 217 amino acids of length were used to determinate the consensus sequence, phylogenetic tree and the 3D M-PDCoV. From the 138 sequences, 112 sequences with 100% similarity were found. Moreover, 26 sequences with at least 94% similarity were found in Asia (China, Thailand, Laos and Vietnam) and one sequence in the USA. These 26 sequences were observed with a different length branch (Figure 1a) and some, grouped together, share the same sequence. Therefore, 16 (black dots, Figure 1a) were selected to compare and analyzed at an amino acid level (Figure 1b). The sequence CHzm2019 from China showed the longest branch in the phylogeny and showed 10 different amino acids (Figure 1b). The phylogenetic analysis showed a high degree of conservation in the PDCoV M protein. Hence, a consensus amino acid sequence was determined (consensus M-PDCoV) from the 138 sequences, to be used in the following analysis.

**Figure 2:**
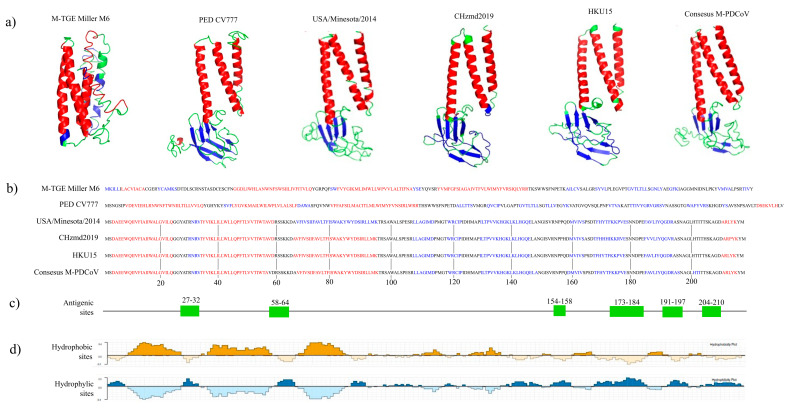
3D predicted model comparison, secondary structures, antigenic sites and surface properties analysis. (**a**) 3D model comparison of consensus M-PDCoV, Chzmd2019, USA/Minnesota292/2014, CHzmd2019, HKU15, M-PED CV777 and M-TGE Miller M6. (**b**) Secondary structure for each predicted model, red: helix, blue: strands, black: coil. (**c**) Six predicted antigenic sites predicted and their positions along the consensus M-PDCoV. (**d**) Consensus sequence surface properties indicate the hydrophobic and hydrophilic sites.

A 3D predicted model comparison analysis was made to determine if the high similarity observed at the amino acid level correlates at a structural level (Figure 2). Using the I-TASSER online server, we obtained the 3D M-PDCoV models for consensus M-PDCoV, USA/Minnesota292/2014, CHzmd2019, HKU15, PED CV777 and TGE Miller M6 (Figure 2a). A C-score of −3.2 was observed for the consensus M-PDCoV, −2.6 for USA/Minnesota292/2014, −2.77 for CHzmd2019, −2.55 for HKU15, 0.42 for PED CV777 and −1.14 for TGE Miller M6. The predicted models were visualized with PyMOL. The comparison analysis shows a similar predicted 3D model for the consensus M-PDCoV, USA/Minnesota292/2014, CHzmd2019 and HKU15. However, a different predicted 3D model was observed for PED CV777 and TGE Miller M6 (Figure 2a). I-TASSER provides which amino acids are alpha-helices, beta-strands and coils in the sequences (Figure 2b). Similar primary structure for the consensus M-PDCoV, USA/Minnesota292/2014, CHzmd2019 and HKU15 were observed. Different secondary structures were observed for PED CV777 and TGE Miller M6 (Figure 2b). Moreover, the percentage of amino acid identity (AAI) was determined. We observed 23.5% of AAI between the consensus M-PDCoV and the CV777 PEDV, 18.89% between the consensus M-PDCoV and TGE Miller M6 and >95% between the consensus M-PDCoV and the others PDCoV (USA/Minnesota292/2014, CHzmd2019 and HKU15). On the other hand, the in silico analysis showed six potential antigenic sites along the consensus M-PDCoV (^27^YATRNR^32^, ^58^VDRSSKK^64^, ^154^RNPPQ^158^, ^173^FKKRVESNNDPE^184^, ^191^QGDRASN^197^ and ^204^TTSKAGD^210^) (Figure 2c). These antigenic sites correlate with hydrophilic sites located on the surface of the protein structure (Figure 2d). On the other hand, no potentially glycosylated sites were predicted using the online NetNGlyc-1.0 software.

To assess the topological similarities between two 3D predicted models, a template modeling score (TM-score) value was determined. A TM-score > 0.7 was observed when comparing the consensus M-PDCoV with CHzmd2019 and HKU15 protein M models, meaning a high similarity between these models. On the other hand, the lowest topological similarities, from 0.1753 to 0.23, were observed when comparing the TGE Miller M6 protein M with the rest of the models. A TM-score value of 0.2 was observed when comparing the PED CV777 protein M model with the rest of the models (Table 1). Therefore, these results indicate the similarity between the consensus M-PDCoV and other PDCoV protein M sequences, and the difference with other swine coronaviruses, such as PEDV and TGEV.

### 3.2. Cloning and Expression of rM-PDCoV

**Figure 3:**
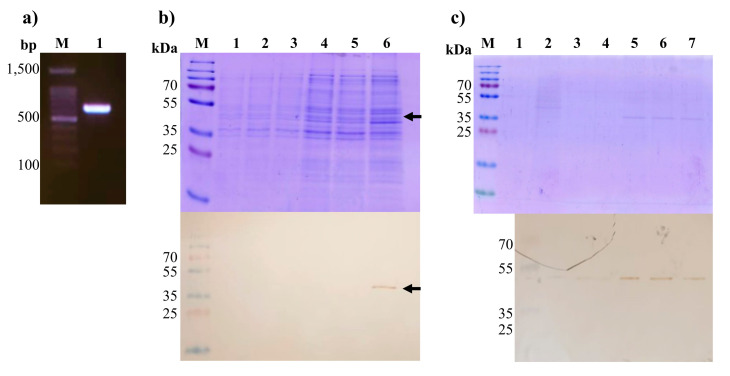
*r*M-PDCoV expression. (**a**) Integrity and weight verification (654 bp) of the PDCoV-M synthetic gene. (**b**) Western blot and SDS-PAGE to verify the *r*M-PDCoV expression. Lines 1 to 3 are samples of the culture media before the *E. coli* BL21 (DE3) disruption. Line 4 is a sample after the *E. coli* BL21 (DE3) disruption. Lines 5 and 6 are samples after centrifuging of the cultures, soluble and insoluble phases, respectively. The *r*M-PDCoV was found in the insoluble phase as inclusion bodies. (**c**) The *r*M-PDCoV confirmation after purification with Ni-NTA agarose column with His-tag affinity. Purified recombinant proteins were observed from elutions 5 to 7. The *r*M-PDCoV is 37.7 kDa of the expected molecular weight.

A synthetic gene (654 bp) was acquired from a commercial supplier using the consensus M-PDCoV determined in this study. Gene integrity and weight were observed in a 1% agarose electrophoresis gel (Figure 3a) with a molecular weight as expected. After cloning the synthetic gene in the pET-SUMO vector, competent *E. coli* BL21 (DE3) cells were made. The pETSUMO expression system produces recombinant proteins fused to the 6his-tag, which allows SUMO protein purification and, in turn, enhances overexpression under IPTG induction.

To determine if the recombinant M-PDCoV (*r*M-PDCoV) were in a soluble or insoluble phase, a Western blot was created using culture media samples after and before cell disruption. Figure 3b shows three samples from an *E. coli* BL21 (DE3) culture media before cell disruption (lines 1 to 3), after cell disruption (lines 4 to 5) and after centrifuging the cell disruption (line 6). The *r*M-PDCoV was observed in line 6 (Figure 3b), indicating that the *r*M-PDCoV is forming inclusion bodies. The band size was 37.7 kDa of the expected molecular weight. After solubilization and purification of inclusion bodies, the *r*M-PDCoV were confirmed from the elutions 4 to 6 in the SDS-PAGE and Western blot (lines 4 to 6) and the molecular weight was in accordance with the expected ~37.7 kDa (Figure 3c).

### 3.3. The rM-PDCoV Immunogenicity Determination in BALB/c Mice

**Figure 4:**
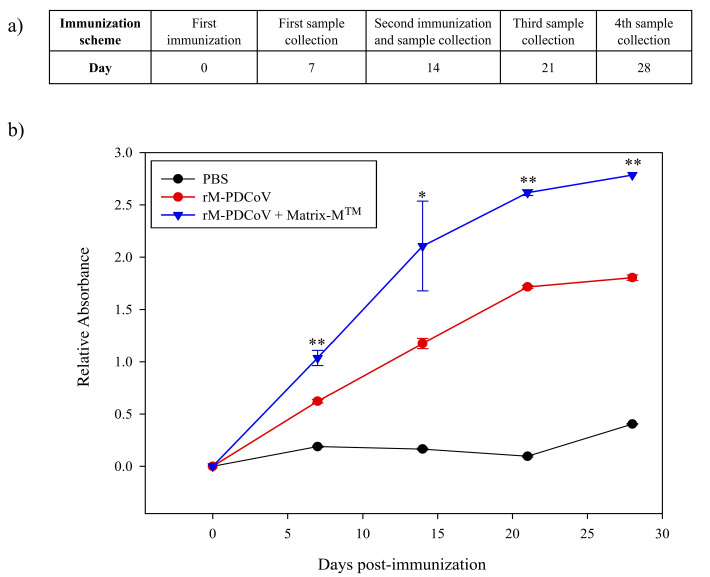
Antigenicity assay. (**a**) Immunization scheme used to evaluate the antibody production. (**b**) Antibody production against *r*M-PDCoV in three BALB/c mice. The blue line indicates the *r*M-PDCoV + Matrix-M^TM^ adjuvant group. The red line indicates the *r*M-PDCoV group. The black line indicates the mouse group immunized with only PBS. Dots and triangles indicated the days of sera collection: 7, 14, 21 and 28. The bars indicated the standard error of mean. The statistical significance was calculated using KlusKal Wallis and Dunn’s test. *p* < 0.05 and *p* < 0.005 is the statistical significance with a 95% confidence interval represented by * and **, respectively.

The *r*M-PDCoV was used to immunize mice according to a specific inoculation scheme (Figure 4a). After the first dose of immunization, we observed an increased level of antibody production in the *r*M-PDCoV- Matrix-M^TM^ group (red line) and *r*M-PDCoV group (blue line), with a statistical significance of *p* < 0.05 (**), respectively. After the second immunization, the antibody production was dramatically higher in both groups, with a statistical significance of *p* < 0.05 (**). Antibody production was maintained until day 28 (Figure 4b). The black line indicates a group of mice immunized only with a PBS buffer.

### 3.4. The Use of rM-PDCoV Antigenic Evaluations Using in Pig Farm Sera Samples

**Figure 5:**
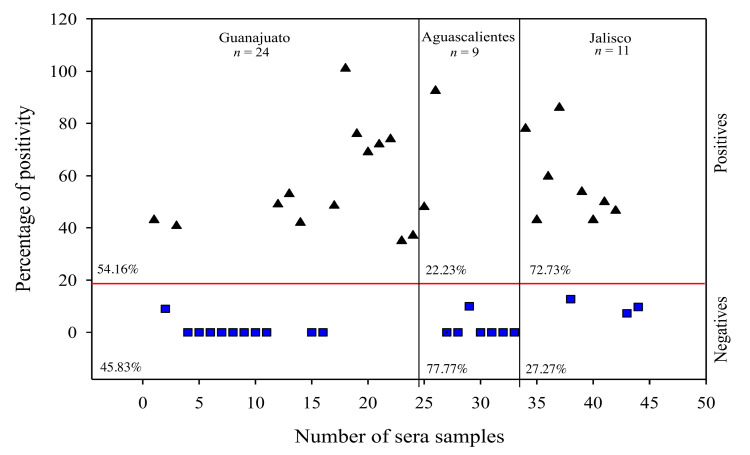
Antigenicity evaluation of the *r*M-PDCoV by an iELISA assay using 44 sera samples from Guanajuato, Aguascalientes and Jalisco. Positive and negative samples are represented by black triangles and blue squares, respectively. The cut-off value is shown by the red line which corresponds to an absorbance of 0.3732 ± 3 SD and 14.3137% PP. Guanajuato: 13 positives (30.23%) and 11 negatives (25.58%); Aguascalientes: 2 positives (4.65%) and 7 negatives (16.28%); Jalisco: 8 positives (18.6%) and 3 negatives (6.98%).

Positive samples were selected based on the cut-off value, which corresponds to an absorbance of 0.3732 ± 3 SD and 14.3137% PP (see Materials and Methods). Then, 44 sera samples from three Mexican states, Guanajuato (*n* = 24), Aguascalientes (*n* = 9) and Jalisco (*n* = 11), located in “El Bajío”, were analyzed by an iELISA assay and the highest absorbance was considered as having 100% PP, which corresponds to an absorbance of 2.3993. A total of 23 positives and 21 negatives sera were observed (Figure 5). From theses, 13 positives (54.16%) and 11 negatives (45.83%) were observed in Guanajuato. In Aguascalientes, two positives (22.23%) and seven negatives (77.77%) were observed. Lastly, eight positives (72.73%) and three negatives (27.27%) were observed in Jalisco. Moreover, it is important to highlight that the 23 positive samples were negative in an iELISA evaluation for NTD-S recombinant protein of PEDV and, in turn, the 21 negative samples were positive to NTD-S recombinant protein of PEDV.

## 4. Discussion

PDCoV is considered to be an emergent disease that causes mortality rates of >40% and frequently co-infects with other SCoVs. Co-infections increase intestinal cell damage and induce a severe infection [16]. In this scenario, an early and suitable diagnosis is of vital importance. After the first report of PDCoV in Mexico [8], no further studies have been developed there. The aim of this study was to evaluate the antigenicity and immunoreactivity of a recombinant M protein of PDCoV (*r*M-PDCoV), developed from a synthetic gene after an in silico analysis, to be used as a potential antigen in a diagnostic system. The essential function in the assembly process of the M protein can explain the lower genetic variability [23,60,61]. The lower variability was confirmed by the phylogeny, with a >94% of similarity among the 138 sequences (ST 1) analyzed. According to the TM-score value, the 3D model of M-PDCoV and USA/Minnesota292/2014, CHzmd2019 and HKU15M models assume roughly the same fold (Figure 2a,b) [53,62]. Together, the phylogeny, 3D model prediction and the structural comparison analysis confirms the lower genetic variability of the M-PDCoV. These analyses indicate that the M-PDCoV is similar to other M protein sequences (USA/Minnesota292/2014, CHzmd2019 and HKU15) reported to have been obtained from naturally infected PDCoV pigs. 

In this study, the *r*M-PDCoV was successfully expressed and purified from a synthetic gene in *E. coli*. BL21 (DE3) using the pETSUMO vector with a ~37.7 kDa expected molecular weight. The use of the pET-SUMO expression vector, which is one of the best vectors for obtaining heterologous proteins destined to develop antigens, was one of the study’s advantages. Furthermore, because of the SUMO tag, huge quantities of functional viral antigens could be produced without affecting their immunogenic and antigenic qualities [55]. Recombinant proteins have frequently been developed [63]; for example, several immune assays based on recombinant proteins (S, M, N) from PEDV have been developed using *E. coli* as an expression system [64,65,66,67]. Likewise, recombinant NP and M proteins of Porcine rubulavirus (PRV) [55], recombinant TGEV N protein with high sensitivity and specificity [68] and, recombinant S, M and N proteins for PDCoV have been developed for antibody detection. Furthermore, no potentially glycosylated sites were predicted by our analysis using the online NetNGlyc-1.0 software; thus, it is considered that using *E. coli* as an expression system to produce the *r*M-PDCoV has no impact on the biological activity, as demonstrated in the antigenicity evaluation described above, and could potentially facilitate the production of diagnostic systems. In addition, a similar result described by Luo S.X. et al. obtained a recombinant no glycosylated M protein in an *E. coli* expression system, which was successfully evaluated in an ELISA assay using >800 sera samples from pig farms [69,70].

PDCoV co-infections with PED have been reported in 54.1% of the cases in Mexico [15]. Therefore, it is possible for a cross-reaction with PED to occur. To avoid the possibility of cross-reaction, amino acid identity (AAI) was determined [54]. The AAI observed for M-PDCoV and CV777 (23.5%) and TGE Miller M6 (18.89%) indicates that the *r*M-PDCoV developed in this study avoids a cross-reaction. These results are similar with those observed by Thachil et al. using the S1 subunit as an antigen [71]. Moreover, Kwonil Jung et al. observed no cross-reaction of the PDCoV USA/IL/2014 isolate with antibodies to either PEDV or TGEV using a PDCoV rabbit antiserum [9,40]. On the other hand, immunogenically. the *r*M-PDCoV successfully stimulated the immune response with detectable antibody production against the *r*M-PDCoV over the weeks after the first immunization dose in BALB/c mice. The antibody response was dramatically higher using a Matrix-M^TM^ as an adjuvant. Matrix-M^TM^ selection was according to our research group [55,72,73]; we have observed that this immunostimulatory complex is an adjuvant that enhances strong immunogenicity using advanced technology in a nanocarrier to deliver antigens [59]. These results are in agreement with previously reported studies, which described antigen enhanced with Matrix-M^TM^ as being highly immunogenic and inducing both antibody and cellular immune responses [57]. In addition, analysis to determine the surface features of the protein and probable antigenic sites (epitopes) that provide information about the biochemical properties of M-PDCoV had not been carried out in previous research where recombinant M-PDCoV protein was produced. In this context, six antigenic sites were found and, from these, four were in the N-terminal region. Viral structural proteins, such as the M protein, possess much higher immunogenicity for T cell responses than nonstructural proteins [74]. For instance, the M protein N-terminal region plays a role as a dominant immunogen for a cellular immune response [36]. Similarly, in alpha, beta and gamma-coronaviruses, the N-terminal region has an interferogenic activity, which produces monoclonal antibodies [75,76]. Furthermore, the *r*M-PDCoV could detect 23 positive (53%) sera samples in the antigenic evaluation among 44 sera samples from three Mexican states (Guanajuato, Aguascalientes and Jalisco) located in the “El Bajío” region, which in turn is ubicated in the central part of Mexico. The presence of PDCoV in the central part of Mexico is consistent with the findings of other researchers, who observed the highest percentage of PDCoV positive samples (15.4%) in the central region [8]. The 53% of positive samples observed in this study suggests that PDCoV is still circulating in the region, infecting pigs. These results suggest that PDCoV could affect swine production, since these three states contribute to >30% of the total swine production in Mexico, in accordance with SIAP, https://nube.siap.gob.mx/cierre_pecuario/b (accessed on 29 March 2023). Therefore, the impact of PDCoV on Mexican pig farms could be greater than expected. Moreover, it has been reported that the main coinfection observed was PDCoV/PEDV, found in 54.1% of the total deltacoronavirus-positive cases [8]. In this context, it is important to highlight that the 23 positives sera observed in this study were negative for PED. Likewise, the 21 observed negatives were positive for PED, both in an iELISA assay for the NTD-S recombinant protein, confirming the non-existence of a cross reaction. Additionally, the cross-reaction was avoided using 37 sera samples from a pathogen free pig farm, without infection outbreaks and without a vaccination history to determinate the iELISA cut-off value.

Altogether, these results indicate that the *r*M-PDCoV developed in this study has the potential to be used as an antigen to detect antibodies against PDCoV in an iELISA in a diagnostic system. Likewise, the *r*M-PDCoV is suitable for detecting positive sera from pig farms, indicating that PDCoV has continued to circulate in Mexico since the first report in 2019. Therefore, the *r*M-PDCoV could be used in futures seroprevalence studies to determine seropositive sera from different regions or states in Mexico. The early diagnosis and disease confirmation of PDCoV must be considered a priority, not only because of the risk it represents in the swine industry, but also because recent findings of PDCoV show an evolutionary change and adaptation leading to human infections [77], indicating the important risk that PDCoV represents. Therefore, systems developed for early detection are of interest and needed. Finally, as a more ambitious and novelty approach, our present study was designed to develop this antigen from 138 M-PDCoV sequences deposited in GeneBank with the goal of using it as a universal antigen capable of detecting PDCoV in samples from different regions or countries. We are currently validating the antigen’s universality.

## 5. Conclusions

The development of the *r*M-PDCoV antigen from a synthetic gene has been suggested as a suitable candidate for enhancing a diagnostic system to detect positive sera from different regions. Furthermore, the *r*M-PDCoV could be used to determine and generate information on the sero-prevalence of PDCoV. Additionally, the *r*M-PDCoV can be used in further studies as an alternative and scalable platform to produce large amounts of a recombinant vaccine in a short time frame.

## Figures and Tables

**Table 1:** Determination of the TM-Score value to assess the topological similarities.

	Consensus M-PDCoV	CHzmd2019	HKU15	M-PED CV777	M-TGE Miller M6	USA/Minnesota292/2014
Consensus M-PDCoV		0.867 *	0.835 *	0.220	0.189	0.797 *
CHzmd2019	0.867 *		0.803 *	0.214	0.190	0.787 *
HKU15	0.835 *	0.803 *		0.217	0.175	0.846 *
M-PED CV777	0.220	0.214	0.217		0.235	0.216
M-TGE Miller M6	0.189	0.190	0.175	0.235		0.181
USA/Minnsota292/2014	0.797 *	0.797 *	0.846 *	0.216	0.216	

* Significant TM-Score values. The value ranges from 0 to 1, where 1 indicates a perfect match between two structures.

## Data Availability

Not applicable.

## Supplementary Materials

The following supporting information can be downloaded at: https://www.mdpi.com/article/10.3390/v15051049/s1, Table S1: Characteristics of the 138 sequences available in GenBank used in this study to obtain the synthetic gene. Figure S1: Western blot analysis to detect positive (*n* = 8) control sera of naturally infected pigs with PDCoV from “El Bajio” pig farm, and negative (*n* = 9) control sera of non-infected pigs from a pathogen-free farm.
