# Supplementary material for: A Candidate Antigen of the Recombinant Membrane Protein Derived from the Porcine Deltacoronavirus Synthetic Gene to Detect Seropositive Pigs
Source: Viruses. 2023 Apr 25;15(5):1049. doi: 10.3390/v15051049 (PMC10222885; doi:10.3390/v15051049)
Supplement: Supplementary file 1 [file viruses-15-01049-s001.zip › Supplementary Figure S1.pdf]

Supplementary material

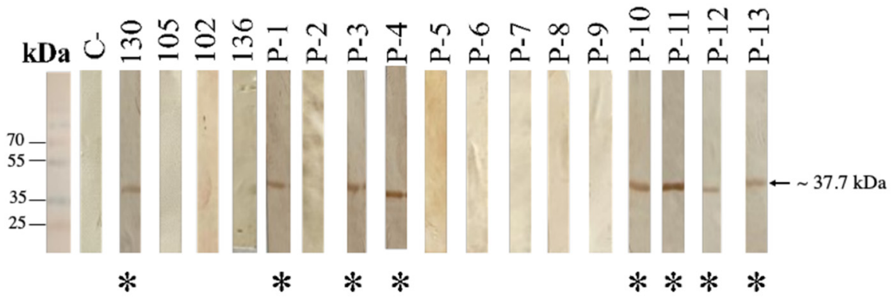

Supplementary Figure S1. Western blot analysis to detect positive (*n* = 8) control sera of naturally infected pigs with PDCoV from "El Bajio" pig farm, and negative (*n* = 9) control sera of non-infected pigs from a pathogen-free farm.
