# Supplementary material for: A Candidate Antigen of the Recombinant Membrane Protein Derived from the Porcine Deltacoronavirus Synthetic Gene to Detect Seropositive Pigs
Source: Viruses. 2023 Apr 25;15(5):1049. doi: 10.3390/v15051049 (PMC10222885; doi:10.3390/v15051049)
Supplement: Supplementary file 1 [file viruses-15-01049-s001.zip › Supplementary Table S1.pdf]

Supplementary material

Supplementary Table S1. Characteristics of the 138 sequences available in GenBank were used in this study to determine the consensus sequence.

No.	Access number	Country	Strain	Year	Isolation source/Host
1	AXP32225.1	China	HNZK-04	2018	Swine
2	AXP32218.1	China	HNZK-02	2018	Swine
3	AXP32232.1	China	HNZK-06	2018	Swine
4	ALA13747.1	China	PDCoV/CHJXNI2/2015	2015	Swine
5	ASK86336.1	China	CH/JXJGS01/2016	2016	Piglet
6	ASK86343.1	China	CH/JXJGS02/2016	2016	Piglet
7	QBQ34483.1	China	CH/JXJGS01/2016	2016	Swine
8	QBQ34490.1	China	CH/JXJGS01/2016	2016	Swine
9	QBQ34497.1	China	CH/JXJGS01/P50	2016	Swine
10	QFU80843.1	China	CHN-JS-2017	2017	*Sus scrofa*
11	ATJ00131.1	China	HB-BD-1	2017	Swine
12	AHM88402.1	USA	USA/IA/2014/8734	2014	Swine
13	AIA60975.1	USA	IL2768	2014	Swine
14	AIA60982.1	USA	SD3424	2014	Swine
15	AIA60989.1	USA	KY4813	2014	Swine
16	AIA60996.1	USA	PA3148	2014	Swine
17	AIA61003.1	USA	NE3579	2014	Swine
18	AIA99520.1	USA	MI6148	2014	Swine
19	AIT11902.1	Korea	KNU14-04	2014	Feces
20	AKA97940.1	USA	USA/IL/2014/026PDV_P11	2015	
21	ALJ32187.1	USA	OH11846	2015	Swine
22	AQT34132.1	Korea	DH1	2017	Swine
23	AQT34138.1	Korea	DH2	2017	Porcine
24	ARJ31776.1	Korea	KNU16-07	2017	Feces
25	QNT22076.1	China	CHzmd2019	2019	*Sus scrofa*
26	AWV96582.1	China	CHN/QH/2017/1	2017	Feces
27	AWV96568.1	China	CHN/GS/2016/2	2016	Feces
28	AWV96575.1	China	CHN/GS/2017/1	2017	Feces
29	AWV96561.1	China	CHN/GS/2016/1	2016	Feces
30	QDF21513.1	China	CHN/Sichuan/2017	2017	Small intestine
31	ALT00614.1	China	LN-DL-2014	2014	Small intestine
32	ATV90796.1	China	CHN-GD16-03	2016	Swine
33	QHI08613.1	China	CH/GX/1468B/2017	2017	intestinal content
34	QEX51275.1	China	CHN-XA18-35	2018	Swine
35	AML40897.1	USA	USA/Minnesota292/2014	2014	Swine
36	ATP16465.1	Vietnam	P29_15_VN_1215	2015	Swine
37	AML40806.1	USA	USA/Indiana453/2014	2014	Swine
38	QDH76190.1	China	CHN-SC2015	2015	*Sus scrofa*
39	QGZ00527.1	China	CHN/Sichuan/2019	2019	Small intestine
40	QDP14513.1	China	CHN/Sichuan/2017_1	2017	Feces
41	QDH76196.1	China	SCNC201705	2017	*Sus scrofa*
42	ATU88975.1	China	CHN-SC2015_2	2016	Swine
43	ASY98689.1	China	CHN-HG-2017	2017	*Sus scrofa*
44	ANK58280.1	Thailand	P24_15_NT1_1215/PDCoV/2015/Thailand	2015	intestine
45	APC23093.1	Thailand	NT1_1215	2015	intestine
46	AMW88199.1	Thailand	TT_1115	2015	intestine
47	APC23094.1	Thailand	BTL_0116	2016	Swine
48	ANK58273.1	Thailand	P2_13_ST2_0313/PDCoV/0213/Thailand	2013	Swine
49	AOM53029.1	Laos	P1_16_BTL_0115/PDCoV/2016/Lao	2016	Small intestine
50	ANK58266.1	Thailand	P1_13_ST1_0213/PDCoV/0213/Thailand	2013	intestine
51	AMN91623.1	Thailand	PDCoV/Swine/Thailand/S5011/2015	2015	Jejunum
52	AMN91629.1	Thailand	PDCoV/Swine/Thailand/S5012/2015	2015	Jejunum
53	AMN91635.1	Thailand	PDCoV/Swine/Thailand/S5013/2015	2015	Feces
52	AMN91641.1	Thailand	PDCoV/Swine/Thailand/S5014J/2015	2015	Jejunum

55	AMN91672.1	Thailand	PDCoV/Swine/Thailand/S5015L/2015	2015	Swine
56	AMN91684.1	Thailand	PDCoV/Swine/Thailand/S5018/2015	2015	Blood
57	AMN91690.1	Thailand	PDCoV/Swine/Thailand/S5019/2015	2015	Blood
58	AMN91696.1	Thailand	PDCoV/Swine/Thailand/S5022/2015	2015	Feces
59	AMN91708.1	Thailand	PDCoV/Swine/Thailand/S5024/2015	2015	Feces
60	AMN91714.1	Thailand	PDCoV/Swine/Thailand/S5025/2015	2015	Feces
61	QBF80903.1	Vietnam	P19_16_VN_0416	2016	intestine
62	QFU80843.1	China	CHN-JS-2017	2017	*Sus scrofa*
63	QCO76965.1	China	Swine/CHN/SC/2018/1	2018	Small intestine
64	QDP14520.1	China	CHN/Sichuan/2018	2018	Small intestine
65	QOP74992.1	China	HeN/swine/2015	2015	Fence
66	ASR75152.1	China	SD	2014	Feces
67	AUH28252.1	China	SHJS/SL/2016	2016	Swine
68	AVZ61084.1	China	CHN-HeB1-2017	2017	*Sus scrofa*
69	QIV13101.1	China	CHN-GX09-2018	2018	Swine
70	QDO79111.1	China	CHN-GX01-2018	2018	Swine
71	QIV13081.1	China	CHN-GX11-2018	2018	Swine
72	QIV13088.1	China	CHN-GX12-2018	2018	Swine
73	QIV13094.1	China	CHN-GX81-2018	2018	Swine
74	AUG59166.1	China	CH/Jiangsu/2014	2014	Feces
75	ATV90800.1	China	CHN-GD16-05	2016	Swine
76	AKC54430.1	China	CHN-AH-2004	2004	Swine
77	APW35736.1	China	HB-BD-2	2016	Swine
78	APG38200.1	China	CHN/Tianjin/2016	2016	Feces
79	AVR48521.1	China	CHN-GD-2016	2016	LLC-PK cells
80	AYU65234.1	China	PDCoV/CHGD/2016	2016	Swine
81	QWE80494.1	Haiti	PDCoV/Haiti/Human/0081-4/2014	2014	*Homo sapiens*
82	QWE80510.1	Haiti	PDCoV/Haiti/Human/0329-4/2015	2015	*Homo sapiens*
83	AQS99155.1	China	CH-01	2016	
84	AUG59158.1	China	CH/Hunan/2014	2014	Feces
85	AKQ63085.1	China	CH/Sichuan/S27/2012	2017	Feces
86	QUM93289.1	China	HNZK-04-P5	2018	Swine
87	QUM93296.1	China	HNZK-04-P15	2018	Swine
88	AKC54444.1	China	CHN-JS-2014	2014	Feces
89	ALD83758.1	China	CH/SXD1/2015	2015	Swine
90	AYP31080.1	China	CHN-HN-1601	2016	Feces
91	AML83918.1	China	CHN-LYG-2014	2014	Feces
92	AML40778.1	USA	USA/Minnesota442/2014	2014	Swine
93	AML40785.1	USA	USA/Minnesota214/2014	2014	Swine
94	AML40883.1	USA	USA/Ohio444/2014	2014	Swine
95	AML40890.1	USA	USA/Ohio445/2014	2014	Swine
96	ANI85827.1	USA	PDCoV/USA/Iowa136/2015	2015	Feces
97	ANI85834.1	USA	PDCoV/USA/Minnesota140/2015	2015	Feces
98	ANI85841.1	USA	PDCoV/USA/Nebraska137/2015	2015	Feces
99	ANI85848.1	USA	PDCoV/USA/Nebraska145/2015	2015	Feces
100	AML40855.1	USA	USA/NorthCarolina452/2014	2014	Swine
101	BAZ95611.1	Japan	HKD/JPN/2016	2016	*Sus scrofa*
102	APC23097.1	Thailand	VN_1_1215	2015	intestine
103	AML40904.1	USA	USA/Iowa459/2014	2014	Swine
104	BBA66307.1	Japan	AKT/JPN/2014	2014	*Sus scrofa domesticus*
105	BBA66328.1	Japan	IWT/JPN/2014	2014	*Sus scrofa domesticus*
106	AML40869.1	USA	USA/Nebraska209/2014	2014	Swine
107	AML40876.1	USA	USA/Nebraska210/2014	2014	Swine
110	AML40862.1	USA	USA/Minnesota159/2014	2014	Swine
111	AML40841.1	USA	USA/Illinois272/2014	2014	Swine
112	AML40848.1	USA	USA/Illinois273/2014	2014	Swine
113	AML40606.1	USA	USA/Arkansas61/2015	2015	Swine
114	AML40792.1	USA	USA/Michigan447/2014	2014	Swine
115	AML40799.1	USA	USA/Michigan448/2014	2014	Swine
116	AML40813.1	USA	USA/Illinois449/2014	2014	Swine
117	AML40820.1	USA	USA/Minnesota/2013	2013	Swine
118	AML40827.1	USA	USA/Minnesota454/2014	2014	Swine
119	AML40834.1	USA	USA/Minnesota455/2014	2014	Swine
120	ASS83088.1	South Korea	KNU16-11	2016	Feces
121	BBA66314.1	Japan	GNM-1/JPN/2014	2014	*Sus scrofa domesticus*
122	BBA66321.1	Japan	GNM-2/JPN/2014	2014	*Sus scrofa domesticus*

123	BBA66335.1	Japan	MYZ/JPN/2014	2014	*Sus scrofa domesticus*
124	BBA66342.1	Japan	OKN/JPN/2014	2014	*Sus scrofa domesticus*
125	BBA66349.1	Japan	YMG/JPN/2014	2014	*Sus scrofa domesticus*
126	QWE80502.1	Haiti	PDCoV/Haiti/Human/0256-1/2015	2015	*Homo sapiens*
127	AWG96867.1	South Korea	KNU16-07-P5	2016	Swine
128	AWG96880.1	South Korea	KNU16-07-P20	2016	Swine
129	QPI70914.1	USA	USA/IL/2014/026PDV_P11_3	2019	Swine
130	AKC54437.1	China	CHN-HB-2014	2014	Feces
131	ALT00617.1	China	SD-QD23-2014	2014	Feces
132	ALT00620.1	China	TJN2-2014	2014	Feces
133	APZ76692.1	China	PDCoV/Swine/Vietnam/HaNoi6/2015	2015	Feces
134	ALT00612.1	China	HLJ-HH-2014	2014	small intestinal
135	ALT00615.1	China	SD-QD3-2014	2014	Boar feces
136	ALS54088.1	China	CHN-HN-2014	2014	*Sus scrofa*
137	AHN16222.1	China	OH1987	2014	Swine
138	YP_009513023.1	China	HKU15	2012	Swine
